# Global Dialysis Perspective: Bangladesh

**DOI:** 10.34067/KID.0000000000000232

**Published:** 2023-10-26

**Authors:** Shubharthi Kar, Md. Foyjul Islam

**Affiliations:** 1 Department of Nephrology, Sylhet MAG Osmani Medical College, Sylhet, Bangladesh; 2 FETP, B (Advance) Fellow, Institute of Epidemiology, Disease Control and Research (IEDCR), Dhaka, Bangladesh

**Keywords:** dialysis, peritoneal dialysis, kidney dialysis

## Introduction

CKD is a global public health concern with increasing prevalence in many countries, including Bangladesh. Studies suggest that in Bangladesh, CKD affects 16%–22% of the population, with 11% of these individuals in stage III–V.^1,2^ In Bangladesh, 200–250 people per million develop end-stage kidney failure (ESKF) each year.^3^ Challenges remain in providing sustainable and effective kidney care because of the financial burden on patients, lack of government subsidies, inadequate number and disproportionate allocation of trained medical professionals, and absence of proper referral and follow-up systems. Dialysis is the primary treatment option for individuals with ESKF in Bangladesh. However, access to dialysis is limited due to the high cost and limited availability of resources.

Despite challenges, the Bangladesh government has implemented policies to increase dialysis access through cost subsidies and new centers, improving the quality of life for ESKF patients. This article outlines dialysis management and financing, the current CKD situation, and the need to address challenges for better renal replacement therapy (RRT) provision and health outcomes in Bangladesh.

## Overview of the Health Care System in Bangladesh

The Ministry of Health and Family Welfare in Bangladesh provides leadership for health and family planning, operating a comprehensive network of medical college hospitals, specialized hospitals, district hospitals, Upazila Health Complexes (subdistrict), Union Health and Family Welfare Centers (Community level), rural dispensaries, and community clinics as part of the Primary Health Care subsystem.^4^ Government has very limited facility for RRT, whereas NGOs, such as Kidney Foundation Bangladesh (KFB) and the Center for Kidney Disease and Urology, offer renal services in the country, with KFB providing 50% of peritoneal dialysis (PD) services and Gonoshasthaya Hospital (a nonprofit hospital) providing cost-effective hemodialysis (HD) services to economically underprivileged patients.^5^

## Bangladesh Perspective of Kidney Disease Management

Bangladesh, a densely populated country with an area of 148,460 km^2^ and a population of 171.3 million, has a gross domestic product of USD 460.2 billion and a gross domestic product *per capita* of USD 2,687, reflecting a medium development level on the basis of the human development index, with a gender inequality index of 0.333%.^6,7^

The country has a total of 262 nephrologists, including pediatric nephrologists. Nephrology in Bangladesh has made significant progress since the country's independence in 1971. In 1973, the country witnessed its first kidney biopsy, and intermittent PD was used to treat ESKF in 1974. HD became a regular form of therapy since 1986. The Bangladesh Renal Association was established in 1977, which helped in the growth of nephrology in Bangladesh. The KFB has introduced voluntary renal registry for ESKF since 2002, and since then, the foundation has been publishing the ESKF registry every year.^1,5^

The incidence of RRT for ESKF was 10,500 (63.75 pmp) in 2019 and 9500 (57.68 pmp) in 2020 (Table 1). HD was the most frequently used RRT in the years 2011–2020, with a peak of 18,922 patients in 2012. However, in recent years, there has been a slight decrease in the number of HD patients, with a low of 16,799 patients in 2020.

**Table 1:** Dialysis management in Bangladesh^1,5–7^

Indicators	Comments
Incidence of RRT in Bangladesh	2019-10,500 (63.75 pmp) 2020-9500 (57.75 pmp)
Prevalence of RRT per million population in Bangladesh	2019–19132 (116.16pmp) 2020-17946 (108.96 pmp)
HD patients per million population	2019–17656 (107.2 pmp) 2020–16500 (100.18 pmp)
Are all dialysis sessions covered by insurance?	No
Dialysis units hospital-based or freestanding?	Hospital-based
Dialysis units for-profit or nonprofit?	For-profit or nonprofit
Reimbursement per dialysis session in USD	No reimbursement facilities available in Bangladesh
Staff who deliver dialysis	Nurses or patient care technicians
Typical patient to RN ratio	5:1
Average length of a dialysis session	4 h
How many times per month are patients seen by a nephrologist during dialysis sessions?	Average three monthly

HD, hemodialysis; RN, registered nurse.

**Figure 1:**
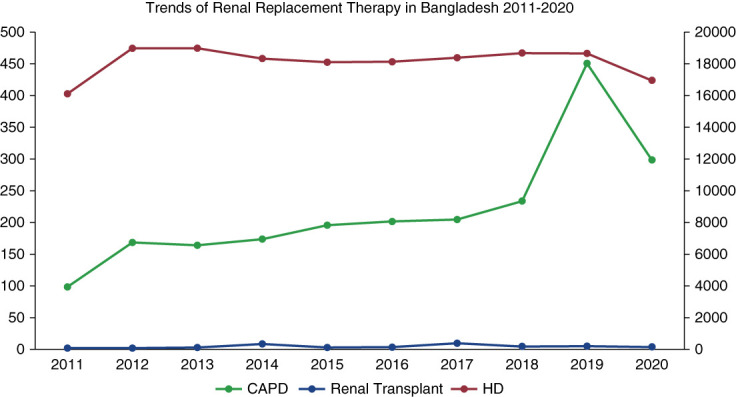
**Trends of RRT in Bangladesh (2011–2020).** CAPD, continuous ambulatory peritoneal dialysis; HD, hemodialysis.

The number of patients on continuous ambulatory PD (CAPD) remained relatively stable over the years, with a peak of 451 patients in 2019. The number of kidney transplantations also remained relatively low, with a peak of 404 transplantations in 2017. Overall, the data suggest a shift toward other forms of RRT in recent years; however, HD remains the most common modality (Figure 1).^1,5,.7^

## HD Practices in Bangladesh

Each year, 35,000–40,000 Bangladeshis develop ESKD.^1^ However, the existing HD facilities can only accommodate 9000–10,000 new patients annually, leaving most ESKF patients without access to RRT.^1^ Chronic glomerulonephritis, diabetic kidney disease, and hypertension are the main causes of ESKF in HD patients.^8^

Most dialysis centers in Bangladesh operate for profit, with costs ranging from US $44–62 per session, compared with nonprofit hospitals, which charge US $12–18.^1^ Limited availability of funds has resulted in the discontinuation of the scheduled dialysis program in government health care institutions in Bangladesh, which previously provided subsidized dialysis at four USD per session for eligible patients selected by a committee. This lack of affordable dialysis highlights the need for increased investment in nonprofit and public health care facilities to promote health equity and improve access to essential medical care.

Dialysis units in Bangladesh are mainly hospital-based, and 170 centers located across the country, primarily in the capital city. There are very few freestanding dialysis centers. Only 84% of HD centers have on-site doctors, and most doctors are medical officers without formal nephrology training. The frequency of HD is mainly twice a week. Most staff who deliver dialysis in Bangladesh are nurses. However, patient care technicians are also used in some facilities. The typical patient-to-nurse ratio varies from institution to institution. However, typical ratio is 5:1 in most dialysis units. In Bangladesh, the average duration of a dialysis session is typically 3–4 hours. Patients in Bangladesh are seen by a nephrologist at 2–3-month intervals during dialysis sessions; however, owing to the shortage of nephrologists in the country, some patients may not have regular access to nephrologists. Most patients initiate HD with noncuffed catheters.^1^ Data are lacking on the proportion of HD patients using arteriovenous fistulas, arteriovenous grafts, and central venous catheters. One single-center study reported a significant proportion (82%) of the 2409 patients initiated HD with noncuffed catheter, whereas only 8% used an arteriovenous fistula.^10^ This study also found that among the patients who used fistulas, most (51%) had brachial fistula, while 38% had radial fistula. The shortage of surgeons and supporting staff is a significant challenge in the context of providing RRT in Bangladesh. Nonetheless, it is clear that improving access to RRT and providing more training for medical staff could help address this pressing issue.

## PD in Bangladesh

PD remains a less common form of dialysis in Bangladesh, with only six centers regularly providing PD services. The number of CAPD patients has increased since 2003 with a total of approximately 450 patients in 2019 and 300 in 2020.^9^ Diabetic nephropathy is the leading cause of ESKF among CAPD patients.^1^ The leading indications for PD are vascular access problems, hemodynamic instability, cardiovascular disease, and older age. Although there has been a gradual improvement in the survival and quality of life of PD patients since 2003, the cost of PD fluid remains a significant obstacle. PD fluid bags are imported and costs approximately 325 taka (US $4.00), and the cost of three exchanges per day is 1000 taka (US $12.5), which is comparable with the cost of HD in for-profit hospitals.^5^ Cost, patient hygiene, peritonitis, and a lack of trained nurses were identified as barriers to increasing PD use in Bangladesh.^11^ Nonetheless, PD offers inherent advantages over HD and has the potential to be a viable alternative if more centers offer the service and the cost is reduced.

## Kidney Transplant in Bangladesh

Kidney transplantation is an established form of RRT in Bangladesh with the first living-related transplant performed in 1982. There are currently ten recognized centers, but only four perform transplants regularly. In 2019 and 2020, there were 1.24 and 0.94 new cases of kidney transplant per million population, respectively.^9^ The most common cause of ESKF in transplanted patients is chronic glomerulonephritis.^1^ Immunosuppression protocols include methylprednisolone and triple therapy. Live unrelated organ donation is banned, favoring deceased transplantation. The law criminalizes organ sales, with penalties of 2–3 years imprisonment or fines.^12^

In Bangladesh, most kidney donors fall within 21–25 years age group and women, specifically mothers, wives, and sisters, accounting for 76% of donors. Studies indicate that donors generally do not experience significant complications postdonation.^11^

Barriers to kidney transplantation in Bangladesh include donor scarcity, low awareness, high costs of immunosuppressive drugs, cultural beliefs, and organ trafficking.^13^ For-profit hospitals charge the highest fees, ranging from USD 6000.00 to 10,000.00 for transplantation procedure. Patients are responsible for postdischarge expenses, such as medications, laboratory tests, and doctor's fees. The cost of immunosuppressive drugs poses a major obstacle, particularly for living-related donors.^5^

## Future Challenges

The future challenges for kidney care in Bangladesh include improving access to dialysis in remote areas, addressing the shortage of trained medical professionals, increasing awareness of kidney disease, and establishing a robust surveillance system. Despite the progress made, there is still much work to be performed to improve the provision of RRT and the health outcomes of those with CKD in Bangladesh.

CKD is a growing public health concern in Bangladesh. Despite the challenges, the progress made by the government and NGOs in increasing dialysis access and improving renal care is commendable. However, there is a need to focus on reducing cost, improving access to dialysis in remote areas, addressing the shortage of trained medical professionals, increasing awareness of kidney disease, and establishing a robust surveillance system. With concerted efforts and investment, it is possible to enhance the provision of RRT and improve the health outcomes of individuals with CKD in Bangladesh.
